# Modeling subcellular specificity in the developing retina

**DOI:** 10.21203/rs.3.rs-3214285/v1

**Published:** 2023-08-08

**Authors:** Ross Perez, Yong Park, Arlene Hirano, Nicholas Brecha, Benjamin Frankfort, Elizabeth Zuniga-Sanchez

**Affiliations:** Baylor College of Medicine; Baylor College of Medicine; David Geffen School of Medicine at UCLA; David Geffen School of Medicine at UCLA; Baylor College of Medicine; Baylor College of Medicine

## Abstract

The precise wiring of the nervous system relies on neurons extending their processes at the right time and place to find their appropriate synaptic partner. The mechanisms that determine when and where neurons extend their neurites during synaptogenesis remains a central question in the field. In the present study, we developed a cell culture system coupled with live imaging to investigate the wiring mechanisms in the developing nervous system. We focused on horizontal cells which are interneurons in the mammalian outer retina known to synapse selectively to distinct photoreceptors. Our data shows cultured horizontal cells extend neurites in a similar manner as *in vivo* with horizontal cells isolated from young mice extending more complex processes compared to those from adult retinas. In addition, horizontal cells cultured alone do not extend neurites and require other retinal cells for neurite extension suggesting that there must be extrinsic cues that promote neurite outgrowth. Moreover, these extrinsic cues do not appear to be solely secreted factors as supernatant from wild-type retinas is not sufficient to promote neurite outgrowth. In summary, we established a new system that can be used to decipher the mechanisms involved in neuronal wiring of the developing central nervous system.

## Introduction

Neural circuit assembly is an intricate and complex process where often a single neuron makes synaptic connections to two distinct targets via different subcellular compartments. This process is referred to as subcellular specificity (reviewed in [1]). To achieve subcellular specificity, neurons must extend their processes at a precise time and location to form connections to their correct synaptic target. The question of how neurons determine when and where to precisely extend their processes to find their partner remains unclear.

**Figure 1:**
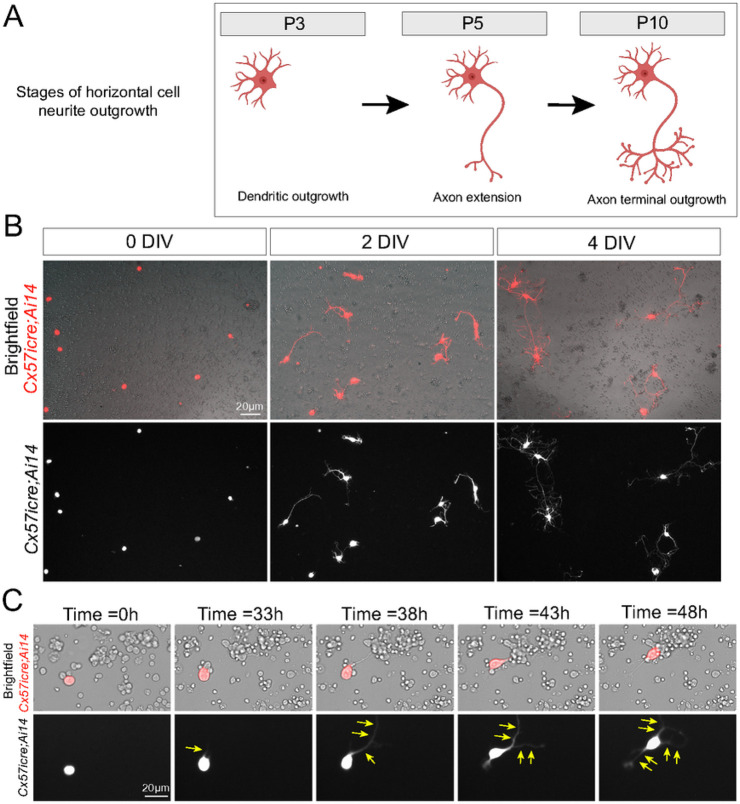
Horizontal cells extend neurites in culture. (A) Schematic drawing of horizontal cells extending neurites at different developmental stages. (B) Horizontal cells (red in top panel; white in bottom panel) extend neurites in a temporal manner as seen in retinal cultures from *Cx57icre;Ai14* transgenic animals at different days in vitro (DIV). (C) Still shots of a live imaging video from a *Cx57icre;Ai14*retinal culture showing a horizontal cell (red in top panel; white in bottom panel) extending neurites (yellow arrows) and making contacts to neighboring cells. Scale bar shown for each figure.

An excellent example of subcellular specificity is found within the mouse outer retina where horizontal cells synapse selectively to the different classes of photoreceptors. The dendrites of horizontal cells synapse selectively to cone photoreceptors whereas the axon terminal synapses to rod photoreceptors [2]. This selective wiring of horizontal cells to photoreceptors is thought to occur at various stages during development. Prior studies have shown that horizontal cells first extend neurites at postnatal (P) day 3, and these neurites preferentially make contacts to cone photoreceptors [3]. Around P5, horizontal cells begin to extend a long process that will eventually become the axon [4], and by P7-9, the axon terminal extends out processes to make contacts to rod photoreceptors [5-7]. See Fig. 1A. These data highlight how the precise timing of neurite outgrowth of horizontal cells is directly linked to their selective wiring. However, the developmental mechanisms that instruct the timing for subcellular specificity of horizontal cells is relatively unknown.

Elucidating the mechanisms of subcellular specificity has been difficult to uncover for several reasons. First, horizontal cells as well as many other neuron types in the central nervous system overlap extensively in both their dendritic and axonal field [4, 8]. Although single labeling approaches such as DiI labeling and AAVs have been used to label single horizontal cells [8, 9], performing these experiments at early developmental time points remains technically challenging. Second, live imaging of horizontal cells has been performed *in vivo* [10]; however, this can only be done for a few hours and not days which is needed to capture the developmental stages of subcellular specificity. And lastly, manipulating the environment to uncover mechanisms of horizontal cell specificity is difficult and cumbersome, heavily relying on transgenic or mutant mouse lines to alter the composition of retinal cell types [11]. To address these issues, we developed a cell culture system coupled with live imaging to begin to decipher the mechanisms underlying horizontal cell specificity in the wild-type retina.

## Results

### Horizontal cells extend neurites in culture similar to in vivo

We first crossed the horizontal cell-specific cre line, *Cx57icre* [12] to the TdTomato fluorescent reporter, *Ai14* [13] to label horizontal cells (referred to as *Cx57icre;Ai14*). Using an optimized protocol to culture retinal neurons [14], we isolated and dissociated retinas from *Cx57icre;Ai14* transgenic animals at various ages and seeded 500 cells per μL. Interestingly, we found horizontal cells isolated from *Cx57icre;Ai14* at P8-10 gradually extend neurites from 0 to 4 days *in vitro* (DIV) as shown in Fig. 1B. This sequential progression of neurite extension is reminiscent to what has been reported *in vivo* as depicted in Fig. 1A. Next, we performed live imaging to capture the dynamic interactions between horizontal cells and other retinal cells in culture. Images were captured every 15 minutes for 48 hours from *Cx57icre;Ai14* retinas isolated at P8 (**Supplemental videos 1,2**). Live imaging data shows that horizontal cells are continuously extending neurites and making contacts to neighboring cells. Figure 1C are still shots of **Supplemental video 1** showing a horizontal cell in culture displaying contact-mediated neurite extension. Together, these findings highlight how horizontal cells extend neurites in cell culture similarly to what has been reported *in vivo* in the mouse retina.

### Horizontal cells from young animals preferentially extend neurites in vitro compared to adult mice

**Figure 2:**
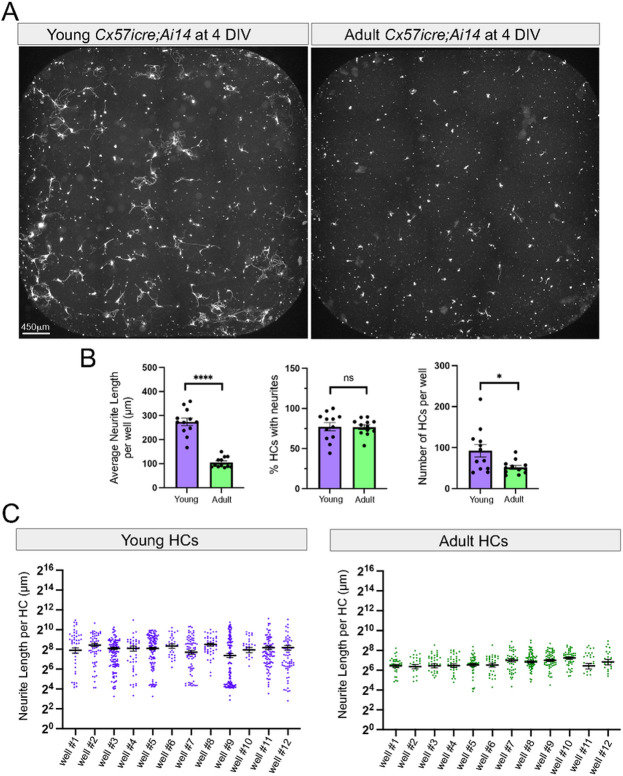
Horizontal cells from young animals extend far more neurites compared to adults. Retinas from *Cx57icre;Ai14* animals were dissociated and cultured for 4 days *in vitro* (DIV). Horizontal cells were fixed and stained with anti-calbindin. (A) Double positive (+calbindin and +TdTomato) cells are shown in white from young animals (P8-10) and display more neurite outgrowth compared to adults (P30). (B) Quantification at 4 DIV of the average neurite length per well, the percentage of horizontal cells with neurites, and the total number of horizontal cells per well in young retinas compared to adults. Data are represented as mean values ±SEM. Statistical significance determined by an unpaired two-tailed Student’s t-test. ns p>0.05, *p<0.05, ****p<0.0001. (C) Neurite length per horizontal cell is shown for each well across different technical (n=6) and biological (n=2) replicates. Mean values ± SEM are plotted for each well. Scale bar shown on figure.

Next, we quantified neurite extension of horizontal cells in our cell cultures using the Imaris confocal software. Retinas were isolated and dissociated from *Cx57icre;Ai14* at P8-10 similar to our live imaging experiments. We refer to these cultures as “Young”. A total of 12 wells from two biological replicates (2–3 animals per experiment) and six technical replicates were used for quantification. We performed antibody staining at 4 DIV using the known horizontal cell marker, anti-calbindin and only used the double + calbindin and + TdTomato cells for further analysis. For quantification, we used the Filament Tracer feature from Imaris to automatically reconstruct individual horizontal cells (+ calbindin and + TdTomato) and measure total neurite outgrowth. Nearly 77% of horizontal cells isolated from young retinas extended neurites with an average neurite length of 273.6 μm ± 15.8 per well (Fig. 2A-B). We then addressed whether horizontal cells isolated at adult stages also have the same capability of extending neurites in cell culture. To test this, we cultured retinal cells from *Cx57icre;Ai14* at P30 (referred to as “Adult”). The overall percentage of horizontal cells extending neurites in adult cultures were not statistically different from young cultures (young: 77.2% horizontal cells with neurites; adult: 76.7% horizontal cells with neurites) even though there were more horizontal cells at 4 DIV in the young cultures compared to the adult as shown in Fig. 2B. However, the average neurite outgrowth per well was significantly lower in the adult (104.9μm ± 6.4 per well) compared to young cultures (Fig. 2A-B). This was consistent across different technical and biological replicates of young and adult cultures as shown in Fig. 2C. Our findings demonstrate that horizontal cells from young animals extend far more neurites in terms of number and length compared to those from adult stages.

### FACS-isolated horizontal cells require other retinal cells to extend neurites

**Figure 3:**
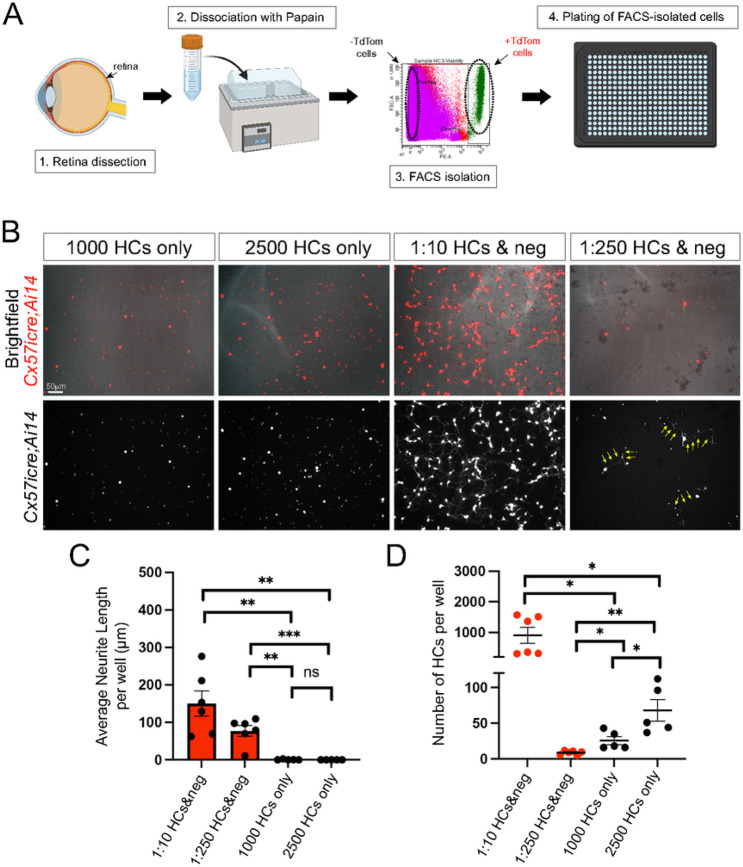
Horizontal cells in culture require the presence of other retinal cells to extend neurites. (A) Schematic drawing of the FACS-isolated horizontal cell procedure. Horizontal cells (+TdTom) and other retinal cells (−TdTom) were isolated from *Cx57icre;Ai14* transgenic animals via FACS and cultured for 4 days *in vitro* (DIV). Horizontal cells were stained with anti-calbindin. (B) Double +calbindin and +TdTom cells are shown in red (top panel) and white (bottom panel). Horizontal cells plated alone at 1000 cells or 2500 cells per well showed no neurite outgrowth. However, horizontal cells plated with other retinal cells (−TdTom) at a ratio of 1:10 or 1:250 showed neurite outgrowth (yellow arrows). (C) Quantification of the sum of neurite length per well is shown for the different experimental conditions. (D) The total number of horizontal cells per well at 4 DIV is shown for each experimental condition. A minimum of three animals were used for each FACS experiment with 3-6 replicates per condition. Data is shown as mean values ± SEM and statistical significance is determined by an unpaired two-tailed Student’s t-test. ns p>0.05, *p<0.05, **p<0.01, ***p<0.001. Scale bar = 50mm.

We then addressed whether horizontal cells in culture extend neurites via intrinsic or extrinsic mechanisms. Specifically, would horizontal cells extend neurites if plated alone (intrinsic), or do they require the presence of other retinal cell types to extend neurites (extrinsic). To test our hypothesis, we isolated horizontal cells via FACS from *Cx57icre;Ai14* animals at P8-10 and plated them in different conditions. For each FACs experiment, we used six retinas from three different transgenic animals. A total of 5–6 wells from two biological replicates (12 retinas from 6 transgenic animals) and 2–3 technical replicates were used for quantification per experimental condition. We gated for single, viable cells and collected both positive and negative TdTomato (TdTom) fluorescently labeled cells as illustrated in Fig. 3A. Next, we plated horizontal cells (+ TdTom) either alone or with other viable retinal cells (−TdTom) at different ratios. We found FACS-isolated horizontal cells plated alone at either 1,000 or 2,500 horizontal cells did not extend neurites after 4 DIV (Fig. 3B-C). However, the same FACS-isolated horizontal cells co-cultured with other retinal cells showed extensive neurite outgrowth (Fig. 3B-C). Horizontal cells (+ TdTom) plated at a 1:10 ratio with other retinal cells (−TdTom) showed the most robust neurite outgrowth (average: 150.1μm ± 33.8 neurite length per well) compared to horizontal cells plated at a 1:250 ratio (average: 76.9μm ± 14.4 neurite length per well). See Fig. 3C. However, the total the number of horizontal cells per well did not correlate with total neurite outgrowth. Cultures that had more horizontal cells per well at 4 DIV such as in “1000 HCs only” and “2500 HCs only” showed poor neurite outgrowth, whereas wells with fewer horizontal cells but plated with other retinal cells (1:250 HCs & neg) had more neurite outgrowth as shown in Fig. 3C-D. These results suggest that there must be extrinsic cues by other retinal cells that promote neurite outgrowth of horizontal cells in culture.

### Secreted factors from retinal cells do not promote neurite outgrowth of horizontal cells in culture

**Figure 4:**
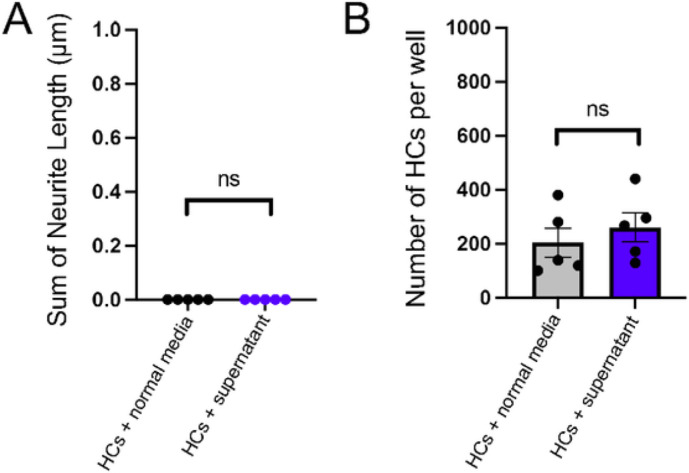
Supernatant alone is not sufficient to promote neurite outgrowth of horizontal cells. (A) Neurites are not observed in horizontal cells cultured with supernatant from 4 days *in vitro* (DIV) retinal cultures or in normal media. (B) The number of horizontal cells at 4 DIV cultured with normal media is not statistically different from those cultured with supernatant. ns rep>0.05.

We next set out to determine if the extrinsic cues that mediates neurite outgrowth is a secreted molecule. To test this, we dissociated retinas from *Cx57icre;Ai14* animals at P8-10 and cultured cells for 4 DIV. After 4 DIV, we collected the media and stored it at −20°C (referred to as “supernatant”). Horizontal cells were isolated via FACS using *Cx57icre;Ai14* transgenic mice at P8-10 and plated at a density of 2,500 cells per well with either normal media or supernatant. A total of 5 wells from three biological replicates (18 retinas from 9 different animals) and 2–3 technical replicates per experiment were used for quantification. Horizontal cells cultured with supernatant did not exhibit neurite outgrowth and did not look different from those cultured with normal media (Fig. 4A). Moreover, the total number of horizontal cells per well at 4 DIV were not statistically different from those cultured in the supernatant (average: 204 ± 54 horizontal cells per well) compared to the normal media (average: 260 ± 54 horizontal cells per well) as shown in Fig. 4B. These results show that the supernatant alone is not sufficient to promote neurite outgrowth of horizontal cells in cell culture.

## Discussion

In summary, we present a new cell culture system that can be used to study the mechanisms underlying horizontal cell specificity during retinal development. We found horizontal cells in culture extend multiple and complex neurites similar to what is seen *in vivo*. However, this was largely dependent on other retinal cells being present in culture as the same number of horizontal cells cultured alone failed to extend neurites. We also found that horizontal cells cultured from young animals (P8-10) preferentially extended neurites compared to adults (P30). These data suggest that there must be developmental mechanisms that promote neurite outgrowth of horizontal cells at early stages; however, these are no longer present in the adult. In addition, we tested whether the signal that promotes neurite outgrowth is a secreted molecule by culturing horizontal cells with 4 DIV supernatant. Our data shows that the supernatant is not sufficient to promote neurite extension suggesting that there must be other cues that induce neurite outgrowth. Taken together, we demonstrate how a new *in vitro* system could be used to decipher the complex cellular and molecular mechanisms involved in neural circuit assembly.

Horizontal cells are known to synapse selectively to the distinct types of photoreceptors via different subcellular compartments. The dendrites of horizontal cells synapse to cone photoreceptors whereas the axon terminal connects to rod photoreceptors [2]. During development, photoreceptors are known to play an important role in horizontal cell morphology. Work by Reese and colleagues showed that the composition of photoreceptors in the retina directly influences dendritic and axonal morphology of horizontal cells [11]. Retinas where rods have been converted to cones though disruption of the rod-specific transcription factor *Nrl* (referred to as “cone-full”) results in more processes in the dendrites of horizontal cells and less in the axon terminal compared to controls. Conversely, ectopic expression of *Nrl* leads to cones being converted to rods (referred to as “rod-full”), and horizontal cells display less processes in the dendrites and more in the axon terminal. These data demonstrate that there must be signaling mechanisms between photoreceptors and horizontal cells that promotes neurite outgrowth of different cellular compartments (i.e. dendrites or axon terminal) during development. Our horizontal cell culture system could be used to identify these mechanisms in a wild-type retina without the need of using transgenic and mutant mouse lines.

Our findings show that supernatant from other cultured retinas is not sufficient to promote neurite outgrowth of horizontal cells in culture. This suggests that there must be additional cues that instruct the extension of neurites from horizontal cells. Cell adhesion molecules are known to be critical players that mediate horizontal cell specificity to the different types of photoreceptors. Netrin-G ligand 2 (NGL2) is a cell adhesion molecule expressed in horizontal cells and localized to the rod synaptic layer [8]. Loss of NGL2 results in loss of synaptic connectivity between rod photoreceptors and the axon terminal of horizontal cells [8]. Similarly, the synaptic cell adhesion molecule (SynCAM1) is highly expressed in rod photoreceptors and loss of SynCAM1 disrupts horizontal cell connectivity [15]. These studies demonstrate that cell adhesion molecules mediate interactions between photoreceptors and horizontal cells which are responsible for proper synaptic connectivity. Thus, there must be cell-to-cell interactions possibly via cell adhesion molecules that mediate neurite extension of horizontal cells in culture.

In addition, we found horizontal cells from both young and adult retinas retain the ability to extend neurites as the same percentage of cells extend neurites in culture. However, the complexity of neurite outgrowth in terms of number and length is significantly lower in adults compared to young animals. These data suggest that there must be signaling factors that promote neurite outgrowth during development, and these are either repressed or no longer present in the adult. Work by Soto and colleagues support this model as re-introduction or overexpression of the cell adhesion molecule, NGL2 in the adult is sufficient to restore and promote neurite outgrowth of horizontal cells *in vivo* [16]. Moreover, recent advancements in single neuron labeling approaches have allowed us to visualize horizontal cells at distinct developmental stages [4]. As the retina develops in a central-to-peripheral wave [17, 18], horizontal cells located in the periphery tend to have multiple axons whereas those located in the center have only one bona fide axon similar to what is seen in the adult retina [4]. This suggests that there must be remodeling that occurs at early developmental stages that ultimately lead to the stereotypic morphology of horizontal cells observed in mature circuits.

In conclusion, we developed a new cell culture system that can be used to begin to decipher the complex mechanisms that mediate neural circuit assembly in the developing retina. Future studies will focus on elucidating the cellular and molecular mechanisms responsible for horizontal cell specificity, with the overall goal of uncovering general principles of neural circuit formation during development.

## Methods

### Isolation and culture of horizontal cells

All methods were performed in accordance with the relevant guidelines and regulations. This study was carried out in accordance with ARRIVE guidelines, and all animal procedures were approved by the Institutional Animal Care and Use Committee (IACUC) of Baylor College of Medicine. *Cx57icre* mice were kindly provided by Nicholas Brecha and crossed to an *Ai14* reporter mouse strain from Jackson Labs (#007914). Developmental time points: P8-10 and P30 mice were used for all experiments. Mice were euthanized with EUTHASOL- pentobarbital sodium and phenytoin sodium solution (ANADA # 200 – 071) by lethal overdose. Retinas were promptly removed after euthanasia, and horizontal cells were either dissociated and plated, or purified via FACS using protocols modified from [14]. Retinas were dissected in oxygenated Ames media (#A1372-25, US Biological) and enzymatically digested with papain (#LS003126; Worthington, Lakewood, NJ) for 12 mins at 37°C followed by trituration with a P-1000 pipette. Dissociated retinal cells were then sieved through a 40μm cell strainer (#352340; Falcon, Corning, NY) to remove tissue clumps resulting in a single cell suspension. Following this procedure, retinal cells were spun down for 8 minutes in a centrifuge at 4°C, 300 x rcf. After removal of the supernatant, retinal cells were resuspended in 4% Ames/BSA (#A-4161, Sigma-Aldrich, St. Louis, MO). Cells were then seeded at a density of 500 cells per uL onto 384-well plates (#781986; Greiner Bio-One, Monroe, SC), coated with poly-D-lysine (#P6407; Sigma-Aldrich, St. Louis, MO, USA) and mouse laminin l (#3400-010-01; Trevigen Gaithersburg, MD). Retinal cells were cultured in a serum-free media with Cytosine β-D-arabinofuranoside (Ara-C) [5μM] (#C6645; Sigma Aldrich, St. Louis, MO) to inhibit the growth of glial cells as described in [14].

### Immunohistochemistry

Horizontal cell cultures were fixed in 4% paraformaldehyde for 15 minutes, followed by subsequent washes in PBS. Cells were then incubated with blocking buffer (10% normal goat serum, 1 % BSA, 0.5% Triton X-100 in PBS) followed by overnight primary antibody staining with Rabbit anti-calbindin (Swant Cat#CB38, RRID:AB_10000340) used at a 1:2000 dilution. After primary antibody incubation, cells were washed 3 times with PBS followed by secondary antibody incubation with Goat anti-Rabbit-647 (Thermo Fisher Cat#A-21244, RRID:AB_2535812) at 1:1000 dilution at 4°C overnight. Cells were then washed 3 times with PBS, stained with DAPI (1:1000), and then each well was filled with Vectashield (Vector Laboratories).

### Imaging Analysis

Retinal cultures were imaged using a Leica DMi8 inverted microscope (Buffalo Grove, IL) and neurite outgrowth of horizontal cells was quantified using the Imaris confocal software version 9.6 (Bitplane, South Windsor, CT, USA). The filament tool in Imaris was used to automatically trace individual neurons with a cell body diameter of 16μm in size and filament thickness of 3μm. The total filament length sum was used to compute the total neurite outgrowth per well based on different experimental conditions. Statistical significance was determined using an unpaired two-tailed Student’s t-test. All statistical analysis were performed using GraphPad Prism version 9 (La Jolla, CA) with p-values given in the text and figure legends.

## Data Availability

All data generated or analyzed during this study are included in this published article and Supplementary files.
